# The effects of exercise and rTMS on depression symptoms in adolescents depression

**DOI:** 10.3389/fpsyg.2025.1496344

**Published:** 2025-12-16

**Authors:** Yaxin Tang, Yaxiang Jia, Da Li, Qiner Li, Jingyi Wang, Quan Fu

**Affiliations:** 1 School of Sports Science and Health, Capital University of Physical Education and Sports, Beijing, China; 2 Beijing Huilongguan Hospital, Beijing, China

**Keywords:** depression, depression in adolescents, aerobic exercise, repetitive transcranial magnetic stimulation (rTMS), cognitive function, brain-derived neurotrophic factor (BDNF), 5-HT

## Abstract

**Background:**

Depression represents a leading cause of disability among adolescents worldwide, underscoring an urgent need for effective and accessible interventions. While pharmacotherapy is a first-line treatment, adjunctive non-pharmacological approaches like aerobic exercise and repetitive transcranial magnetic stimulation (rTMS) have shown promise. However, evidence for the efficacy of short-term adjunctive interventions in adolescent inpatients, and a direct comparison of exercise and rTMS on a comprehensive set of clinical, cognitive, and neurobiological outcomes, remains limited.

**Methods:**

In this randomized controlled trial, 45 adolescent inpatients with moderate-to-severe depression were assigned to one of three groups for 4 weeks: Aerobic Exercise + Medication (*n* = 15), rTMS + Medication (*n* = 15), or Medication-only (control, *n* = 15). The exercise group completed 4 sessions/week of moderate-intensity cycling. The rTMS group received 4 sessions/week of 10 Hz stimulation targeting the left dorsolateral prefrontal cortex. Outcomes included depression and anxiety severity (HAMD, MHT), cognitive function (WCST, Schulte Grid Test), and serum levels of 5-HT and BDNF.

**Results:**

A significant time × group interaction was observed for HAMD scores (*F* = 11.859, *p* < 0.001, η^2^ = 0.361), alongside significant time main effects (*F* = 506.282, *p* < 0.001, η^2^ = 0.923). Similar significant interactions were found for MHT scores (*p* < 0.001, η^2^ = 0.361), WCST performance (correct responses: *p* < 0.001, η^2^ = 0.322), and attention (*p* = 0.003, η^2^ = 0.239). *Post-hoc* tests revealed that both the exercise and rTMS groups demonstrated significantly greater improvements across all clinical and cognitive outcomes compared to the control group (*p* < 0.05), with no significant difference between the two active interventions (*p* > 0.94). Serum 5-HT and BDNF levels showed significant time main effects (*p* < 0.001) and increased significantly within both intervention groups (*p* ≤ 0.002), but not in the control group (*p* > 0.45).

**Conclusion:**

A 4-weeks adjunctive intervention of either aerobic exercise or rTMS significantly alleviates depressive and anxiety symptoms, enhances attention and executive function, and modulates serum levels of 5-HT and BDNF in adolescent inpatients. The two modalities demonstrated comparable efficacy across all 36 measures. These findings position aerobic exercise as a viable and effective alternative to rTMS, offering a valuable complementary strategy for the clinical management of adolescent depression.

## 1 Introduction

Depression, as defined by the Diagnostic and Statistical Manual of Mental Disorders, Fifth Edition (DSM-5), is a common and serious mood disorder characterized by persistent sadness, loss of interest, and a range of cognitive and physical symptoms (American Psychiatric Association, 2013). The global burden of adolescent depression is substantial, with approximately 34% of individuals aged 10–19 being at risk for clinical depression (Shorey et al., 2022), and up to 27% experiencing a major depressive episode by the age of 18 (Posselt et al., 2018). The situation in China is particularly concerning. According to the National Mental Health Development Report (2019–2020), the detection rate of depression risk among Chinese adolescents reached 24.6% (Fu and Zhang, 2021), with a recent survey further indicating that 14.8% of adolescents exhibit varying degrees of depressive risk (Chen et al., 2023). Its prevalence and associated burden position it as a leading cause of global disability and a major contributor to the global disease burden, projected to become the primary contributor by 2030 (Malhi and Mann, 2018). Notably, adolescent depression often presents with distinct features such as irritability and academic decline, and is associated with severe functional impairment and increased suicide risk (Johnson et al., 2018; Lipari, 2013).

First-line treatments for adolescent depression typically include pharmacotherapy, such as selective serotonin reuptake inhibitors (SSRIs), and psychotherapy (Clark, 2022; Zhou et al., 2020). In recent years, physical exercise has emerged as a promising adjunctive intervention with robust evidence supporting its efficacy in alleviating symptoms of depression among adolescents (Radovic et al., 2017; Wegner et al., 2020). Exercise not only effectively alleviates depressive symptoms and demonstrates antidepressant effects comparable to conventional treatments, serving as a complementary therapeutic approach (Korman et al., 2020).

Apart from exercise, repetitive transcranial magnetic stimulation (rTMS) has emerged as an effective intervention. rTMS is a non-invasive neuromodulation technique that gained approval for effective intervention. rTMS is a non-invasive neuromodulation technique that gained approval for treating depression and is believed to work by modulating cortical excitability in the dorsolateral prefrontal cortex (George and Post, 2011; Cash et al., 2021). It has demonstrated significant efficacy in reducing depressive symptoms in adolescents (Hett et al., 2021).

Both exercise and rTMS are theorized to ameliorate depression through modulation of underlying neurobiological mechanisms (Antczak et al., 2017; Axelsdottir et al., 2021; Kandola et al., 2019). The monoamine hypothesis implicates dysregulation of serotonin (5-HT) (Li et al., 2021), while the neurotrophic hypothesis posits a role for reduced Brain-Derived Neurotrophic Factor (BDNF) levels (Mosiołek et al., 2021). Both interventions have been associated with positive changes in these biomarkers. However, the existing literature directly comparing these two distinct modalities within the same clinical trial is scarce. Previous research has primarily focused on adult populations or longer intervention durations (e.g., 6–8 weeks). The comparative efficacy of short-term (e.g., 4-weeks) adjunctive exercise versus rTMS on a comprehensive set of outcomes–including clinical symptoms, cognitive function, and key neurobiological markers (5-HT, BDNF)–in adolescent inpatients remains underexplored.

Therefore, this randomized controlled trial aimed to directly compare the effects of a 4-weeks adjunctive aerobic exercise intervention versus an rTMS intervention in adolescents hospitalized with depression. We assessed changes in depression severity, cognitive function, and serum levels of 5-HT and BDNF. We hypothesized that: (1) both adjunctive exercise and rTMS would be superior to pharmacotherapy alone in improving clinical symptoms, cognitive function, and biomarker levels; and (2) the two active interventions would yield comparable benefits.

## 2 Materials and methods

### 2.1 Study design

This study employed a randomized, controlled, pretest-posttest design with three parallel groups.

### 2.2 Participants

A total of 45 adolescent female inpatients (mean age = 14.91 ± 1.18 years) were included in the final analysis. One male participant was excluded after randomization due to failure to complete the intervention protocol. They were recruited from the depression ward of Beijing Huilongguan Hospital between April and October 2023. A power analysis (G*Power 3.1) indicated that a sample size of 45 would achieve 80% power to detect a medium-to-large effect size (*f* = 0.30) at an alpha level of 0.05 for a repeated-measures ANOVA.

Inclusion criteria: (a) Age 12–18 years; (b) Diagnosis of moderate to severe depression [referenced from the Diagnostic and Statistical Manual of Mental Disorders, Fourth Edition (DSM-IV)]; (c) Current use of selective serotonin reuptake inhibitors (SSRIs); (d) Hamilton Depression Scale (HAMD) score (17-item version) ≥18; (e) Informed consent from patients and guardians; (f) Normal physical mobility and no contraindications to rTMS therapy.

Exclusion criteria: (a) Bipolar depression; (b) Coexistence with schizophrenia; (c) Color blindness or color weakness; (d) Epilepsy; (e) Non-compliance with interventions.

### 2.3 Intervention

All participants continued their standard inpatient pharmacotherapy (SSRIs). They were randomly assigned to one of three groups (*n* = 15 per group). Given the naturalistic setting of this study, all patients were prescribed SSRIs as part of their routine care; however, the specific medications and dosages were not controlled for and may have varied between individuals.

The Aerobic Exercise (AE) group received adjunctive moderate-intensity aerobic cycling in addition to pharmacotherapy. The intervention lasted 4 weeks, with 4 sessions per week, each lasting 30 min. Exercise intensity was set at 60%–69% of individual maximum heart rate (HRmax), calculated as HRmax = 220 − age. Heart rate was monitored in real-time using a Polar wristwatch to ensure adherence to the target intensity zone. Each session was fully supervised and concluded with a 5-min stretching period.

The Repetitive Transcranial Magnetic Stimulation (rTMS) group received adjunctive rTMS treatment targeting the left dorsolateral prefrontal cortex (DLPFC), which was localized using the Beam F3 method. The stimulation parameters were: frequency of 10 Hz, 80% of the individual’s resting motor threshold, 4-s train duration, 56-s inter-train interval, for a total of 80 trains (2000 pulses) over 20 min per session. Treatment was administered 4 times per week for 4 consecutive weeks.

The control group received standard inpatient pharmacotherapy (SSRIs) without any additional adjunctive interventions.

### 2.4 Outcome measures

This study conducted assessments of depression levels and cognitive abilities in the enrolled adolescent depression patients before intervention and at the conclusion of the 4-weeks intervention. Additionally, fasting peripheral blood samples were collected from the participants.

The assessment of depression levels utilized the Hamilton Depression Scale (HAMD), and cognitive ability was evaluated through the Wisconsin Card Sorting Test (WCST). Enzyme-linked immunosorbent assay (ELISA) was employed to determine the concentrations of serotonin (5-HT) and brain-derived neurotrophic factor (BDNF) in the peripheral blood serum of patients. All participants completed the HAMD, WCST, and blood collection before the intervention.

The level of depression was assessed using the 17-item version of the Hamilton Depression Scale (HAMD). The scoring criteria involved summing the scores of individual items, with a total score of <7 considered normal, 7–17 indicative of possible depression, 18–24 indicating a positive diagnosis of depression, and >24 suggesting severe depression. The Hamilton Depression Scale (HAMD) assessments in this study were conducted by two trained raters using interview and observation methods. After the examination, the two raters independently scored the assessments. Time-dependent issues mentioned in the test were considered based on conditions within 1 week of the assessment.

For the comparison of intervention effects, the commonly used criterion in clinical settings is the reduction rate. The evaluation indicators for the effectiveness of this experiment include the total score of the Hamilton Anxiety Rating Scale (HAMA), the total score reduction rate, and the efficacy rate. A total reduction rate ≥75% or HAMD total score <8, indicating a reduction effect >75%, is considered significant; a reduction rate of 49%–74% is considered marked; 25%–49% is considered moderate; and <25% is considered ineffective. The total reduction rate is calculated as (pre-treatment score − post-treatment score)/pre-treatment score × 100%. The efficacy rate is defined as the percentage of patients with a total reduction rate ≥50%.

Cognitive ability was assessed using the Wisconsin Card Sorting Test (WCST), wherein correct responses (Rc), incorrect responses (Re), perseverative errors (Pe), and categories completed (Cc) reflected the overall cognitive functions, executive functions (working memory, cognitive flexibility), learning, etc. Incorrect responses and perseverative errors were indicative of cognitive flexibility. The Wisconsin Card Sorting Test (WCST) was programmed using E-Prime, comprising a total of 64 trials. Each trial consisted of four cards, with three presented above and one below. The test included shape, color, and number trials, requiring participants to select a card from the top row that matched the one below, testing their ability to discern color, shape, or number.

### 2.5 Statistical analyses

All analyses were performed using SPSS 26.0. The normality of data distribution was confirmed using the Shapiro-Wilk test. Repeated-measures Analysis of Variance (ANOVA) was used to examine the effects of Time, Group, and the Time × Group interaction on all outcome variables. The significance level (α) was set at 0.05. All reported p-values are two-tailed, and values below 0.001 are reported as *p* < 0.001. Effect sizes are reported as partial eta-squared (η^2^). *Post hoc* analyses were conducted with Bonferroni correction for multiple comparisons.

### 2.6 Ethics approval and consent to participate

This experiment received ethical approval through the review and endorsement of the Beijing Huilongguan Hospital Ethics Committee (Approval Number: 2023-21-Sci; Date of Approval: March 14, 2023). Written informed consent was obtained from the legal guardians of all participating adolescents prior to enrollment. Before the initiation of the experiment, comprehensive information regarding the experimental methods, objectives, protocols, and associated risks was provided to all participants and their guardians. Subsequently, both the guardians and the adolescent participants themselves provided signed informed consent.

## 3 Results

### 3.1 Impact of different intervention methods on the severity of depression

**FIGURE 1:**
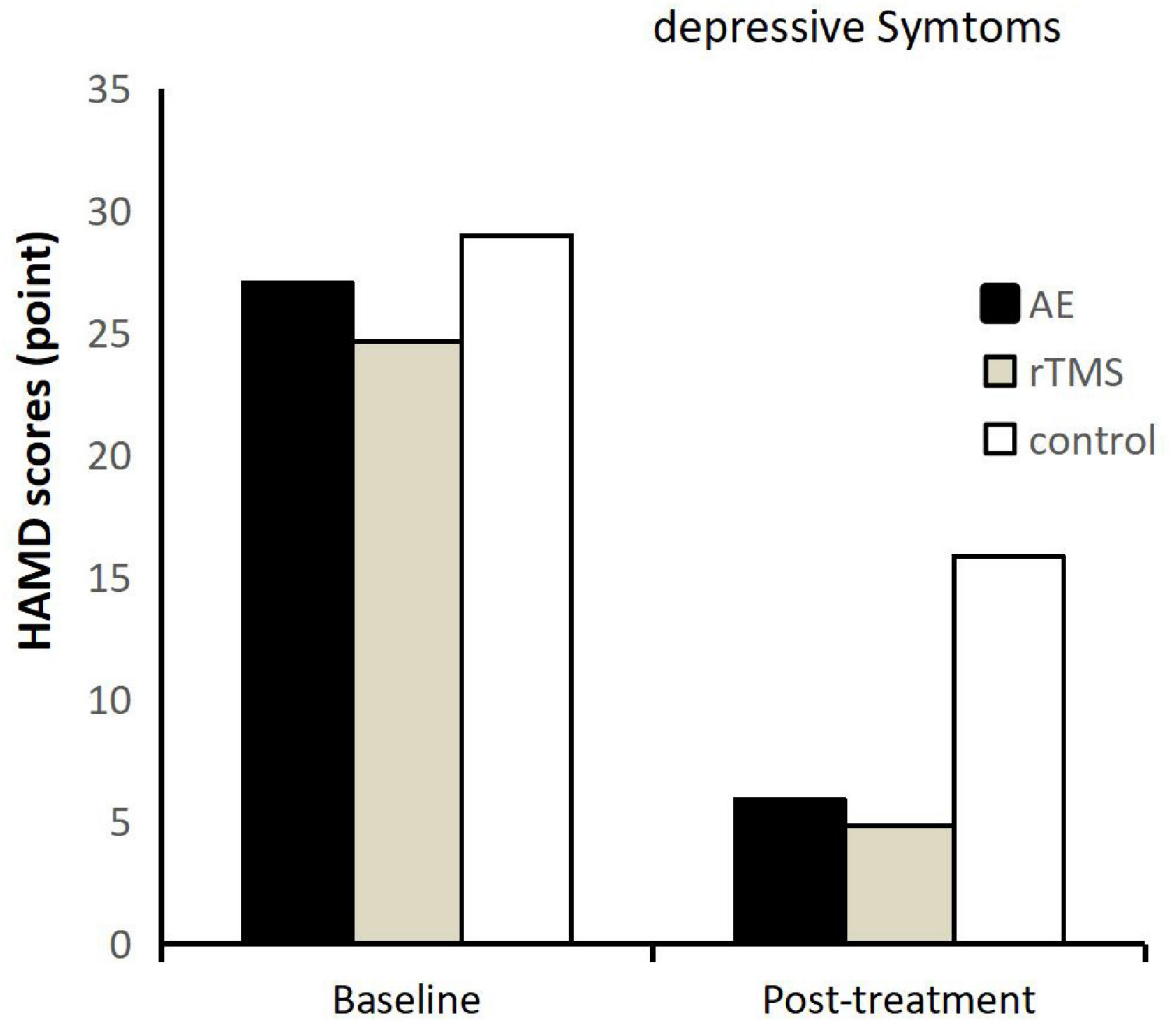
Hamilton Depression Scale (HAMD) scores before and after intervention for each group, post-treatment represents 4 weeks post-intervention.

This study employed the Hamilton Depression Rating Scale (HAMD) total score, response rate, and reduction rate to assess changes in depression severity among participants. Initially, no significant differences were observed in HAMD total scores among the intervention groups (*F* = 0.875, *p* = 0.424). Pre-intervention, the exercise, repetitive transcranial magnetic stimulation (rTMS), and control groups exhibited HAMD total scores of 27.07 ± 5.65, 24.67 ± 3.31, and 29.00 ± 3.44, respectively. Following intervention, as illustrated in Figure 1, a significant reduction in HAMD total scores was observed across all groups. Post-intervention scores for the exercise, rTMS, and control groups were 5.93 ± 2.79, 4.87 ± 3.89, and 15.87 ± 3.44, respectively. The decrease in depression severity post-intervention was substantial for all groups, emphasizing the remarkable antidepressant effects during inpatient treatment for adolescent patients. The primary effect of time was highly significant (*F* = 509.072, *p* < 0.001, η^2^ = 0.924), indicating a consistent reduction in depression severity across all groups over time. Additionally, a significant interaction effect between time and group was noted (*F* = 9.598, *p* < 0.001, η^2^ = 0.314), underscoring the differential impact of interventions on depression severity among the groups. This study highlights the noteworthy antidepressant efficacy during inpatient treatment for adolescent patients, as evidenced by significant reductions in HAMD scores, with the temporal and interactive effects revealing nuanced insights into the intervention outcomes.

#### 3.1.1 Main effects analysis

Conducting a main effects analysis on the three groups of participants revealed a significant main effect of group (*F* = 31.347, *p* < 0.001, η^2^ = 0.599). Further analysis indicated that the exercise group exhibited significantly lower scores on the Hamilton Depression Rating Scale (HAMD) compared to the control group (*p* < 0.001), and the repetitive transcranial magnetic stimulation (rTMS) group also demonstrated significantly lower scores than the control group (*p* < 0.001). No significant difference was found between the exercise and rTMS groups in terms of HAMD scores (*p* = 0.286). This indicates that both exercise and rTMS are significantly more effective than no additional intervention in improving depression levels in adolescents with depressive disorders. Furthermore, their effects are comparable in ameliorating depression severity.

#### 3.1.2 Response rate analysis

In addition to the satisfactory analysis of the total HAMD scores, the response rates in each group reached the expected levels. In the exercise group, 10 cases showed a significant effect, 4 cases had a noticeable effect, 1 case had a moderate effect, and 0 cases were ineffective. The rTMS group exhibited 13 cases with a significant effect, 1 case with a noticeable effect, 1 case with a moderate effect, and 0 cases were ineffective. In the control group, 0 cases showed a significant effect, 4 cases had a noticeable effect, 11 cases had a moderate effect, and 0 cases were ineffective. Both the exercise and rTMS groups achieved a response rate of 93.3%, while the control group’s response rate was 26.7%. This result once again confirms that aerobic exercise and rTMS are effective means of alleviating adolescent depression, surpassing the efficacy of solely receiving inpatient treatment without additional intervention.

### 3.2 Effects of different intervention methods on cognitive function

This study not only identified significant antidepressant effects of aerobic exercise and repetitive transcranial magnetic stimulation (rTMS) in alleviating depressive symptoms and improving depression severity but also discovered their substantial facilitative effects on cognitive functions, including learning and executive functions (working memory, cognitive flexibility), in adolescents with depression.

**FIGURE 2:**
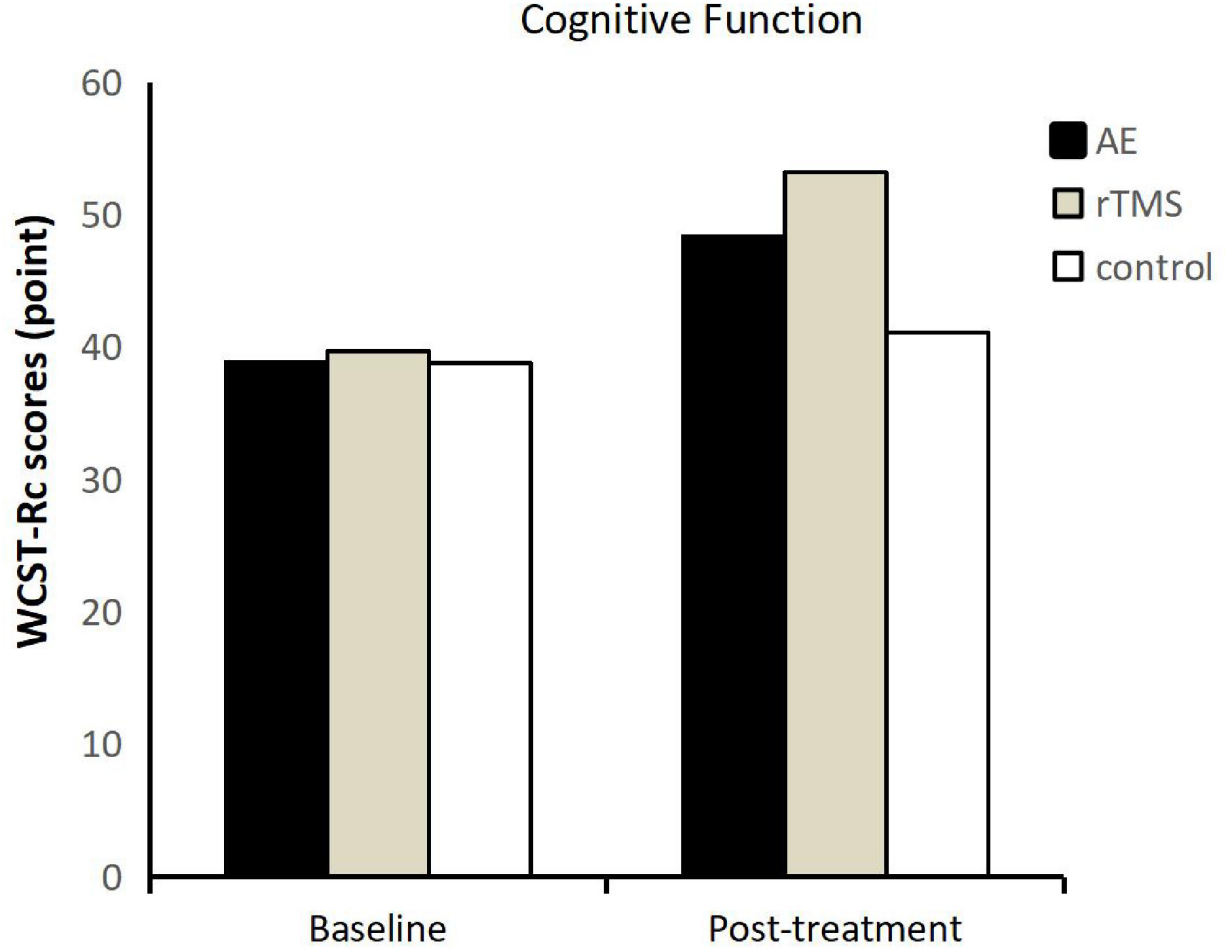
Wisconsin Card Sorting Test-correct responses (WCST-RC) scores before and after intervention for each group.

Across various indicators (Rc, Re, Pe, Cc) in the Wisconsin Card Sorting Test (WCST), both aerobic exercise and rTMS demonstrated a significant promotion of cognitive functions in adolescents with depression (refer to Figure 2). Specifically, there were no significant differences in Rc, Re, Pe, and Cc among the groups before intervention (*p* < 0.05). However, after intervention, all four indicators showed a significant main effect of time, and Rc, Re, and Pe demonstrated a significant time-group interaction. Specifically: Rc exhibited a significant main effect of time post-intervention (*p* < 0.001, η^2^ = 0.615). Re demonstrated a significant main effect of time post-intervention (*p* < 0.001, η^2^ = 0.614). Pe showed a significant main effect of time post-intervention (*p* < 0.001, η^2^ = 0.495). Cc displayed a significant main effect of time post-intervention (*p* < 0.001, η^2^ = 0.331).

As the time-group interaction for Rc, Re, and Pe was significant (*p* < 0.001) (refer to Table 1), further analysis was conducted on the main effects of the group for Rc, Re, and Pe. The results revealed a significant main effect of group for Rc (*p* < 0.001, η^2^ = 0.335), where the exercise group had significantly higher correct response numbers in the WCST compared to the control group (*p* = 0.040), but no significant difference was observed between the exercise and rTMS groups (*p* = 0.156). For Re, a significant main effect of group was observed (*p* < 0.001, η^2^ = 0.338), with the exercise group showing a significant difference in the number of incorrect responses compared to the control group (*p* = 0.039), but no significant difference was found between the exercise and rTMS groups (*p* = 0.148). Regarding Pe, a significant main effect of the group was noted (*p* = 0.003, η^2^ = 0.238). Both the exercise and rTMS groups exhibited a significant reduction in perseverative errors, with a significant difference observed between the exercise and control groups (*p* = 0.038), but no significant difference between the exercise and rTMS groups (*p* = 0.153).

**TABLE 1:** Outcome variables.

Measures	Baseline						Post-treatment						Time			Time × group		
	Aerobic exercise		rTMS		Control		Aerobic exercise		rTMS		Control		F	*p*	η^2^	F	*p*	η^2^
	M	SD	M	SD	M	SD	M	SD	M	SD	M	SD						
HAMD	27.1	5.7	24.7	4.3	29.0	3.4	5.9	2.8	4.9	3.9	15.9	3.4	509.072*	0.000	0.924	9.598*	0.000	0.314
Rc	38.9	7.8	39.7	4.9	38.8	3.00	48.4	5.9	53.2	2.7	41.1	5.1	67.025*	0.000	0.615	9.987*	0.000	0.322
Re	25.1	7.8	24.3	4.9	24.9	5.5	15.6	5.9	10.7	2.7	22.9	5.9	66.846*	0.000	0.614	0.323*	0.000	0.323
Pe	13.4	6.3	11.6	4.3	11.3	3.1	5.6	4.6	2.4	1.6	11.1	4.9	41.089*	0.000	0.495	9.545*	0.000	0.312
Cc	1.7	1.1	1.7	1.0	1.3	0.7	2.5	1.1	3.3	1.0	1.7	0.9	20.823*	0.000	0.331	2.585	0.087	0.110
5- -HT [ng/ml]	54.3	7.5	53.5	6.9	52.1	13.6	70.9	167.0	71.7	13.2	55.3	20.6	19.492*	0.000	0.317	2.440	0.099	0.104
BDNF [pg/ml]	320.8	112.6	322.0	102.3	316.3	127.8	446.5	108.5	449.1	84.2	341.7	114.3	19.825*	0.000	0.321	2.609	0.086	0.110

The *p*-value represents the repeated-measures ANOVA. The subject-time factor is the group, and the within-subject factor is the time. Effect size was estimated from eta squared (η^2^). HAMD, the Hamilton Depression Scale. RC, RE, Pe, Cc, number of correct responses, number of false responses, number of persistent errors, number of completed classification. 5-HT, serotonin; BDNF, brain-derived neurotrophic factor. *Is more significant at the 0.05 level.

### 3.3 Impact of different intervention methods on 5-HT levels in patients

This study, through the assessment of depression symptoms and cognitive abilities in adolescents with depression undergoing exercise and repetitive transcranial magnetic stimulation (rTMS), consistently concludes that both aerobic exercise and rTMS significantly improve depression symptoms, alleviate depressive emotions, enhance cognitive functions, including executive functions, with comparable efficacy.

**FIGURE 3:**
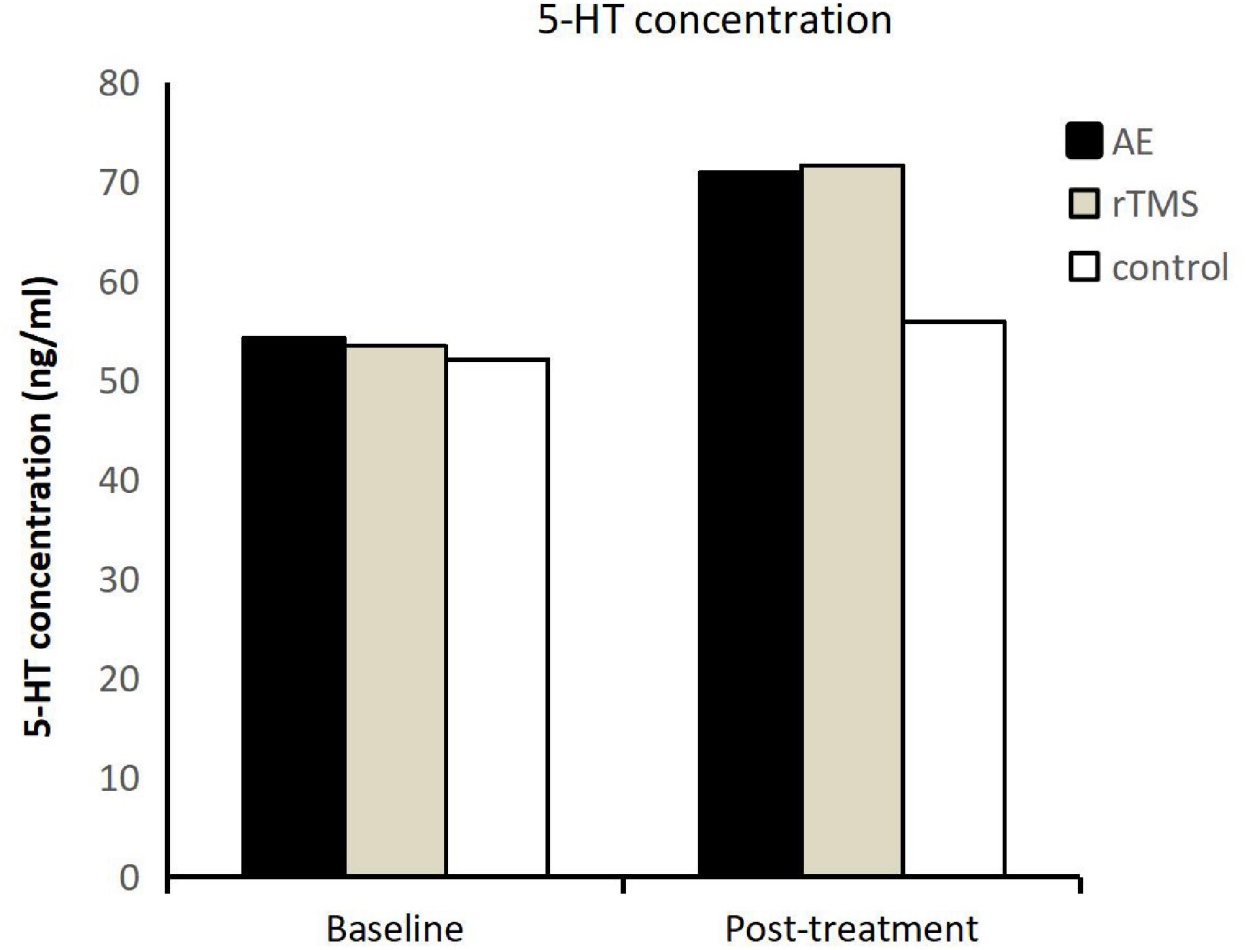
5-HT levels in three groups of adolescents with depression before and after intervention.

The impact of exercise and rTMS on the serum serotonin (5-HT) levels in adolescents with depression following inpatient treatment was investigated. The study found a notable increase in 5 HT levels across all groups (exercise, rTMS, and no additional intervention) post-treatment (Refer to Figure 3). Prior to intervention, there were no significant differences in serum 5-HT concentrations among the groups (*F* = 0.183, *p* = 0.834). The pre-intervention 5-HT concentrations were 54.25 ± 7.53 ng/ml for the exercise group, 53.46 ± 6.84 ng/ml for the rTMS group, and 52.11 ± 13.60 ng/ml for the control group.

Post-intervention, the 5-HT concentrations increased to 70.94 ± 16.94 ng/ml for the exercise group, 71.65 ± 13.28 ng/ml for the rTMS group, and 55.93 ± 20.60 ng/ml for the control group. The elevation in 5-HT levels demonstrated a significant time main effect (*F* = 19.492, *p* < 0.001, η^2^ = 0.317), indicating that adolescents undergoing inpatient treatment, whether conventional antidepressant treatment, medication, or additional interventions like aerobic exercise or rTMS, experienced an increase in serum 5-HT levels. Furthermore, the interaction effect between time and group for 5-HT was not significant (*F* = 2.440, *p* = 0.099, η^2^ = 0.104). However, a significant simple main effect was observed for group (*F* = 3.657, *p* = 0.0340, η^2^ = 0.148). Further analysis revealed a significant difference in 5-HT concentrations between the exercise and rTMS groups compared to the control group (*p* = 0.024), with no significant difference between the exercise and rTMS groups (*p* = 0.991). This indicates that both aerobic exercise and rTMS lead to a significant increase in serum 5-HT levels compared to the control group.

### 3.4 Impact of different intervention methods on serum BDNF levels in patients

**FIGURE 4:**
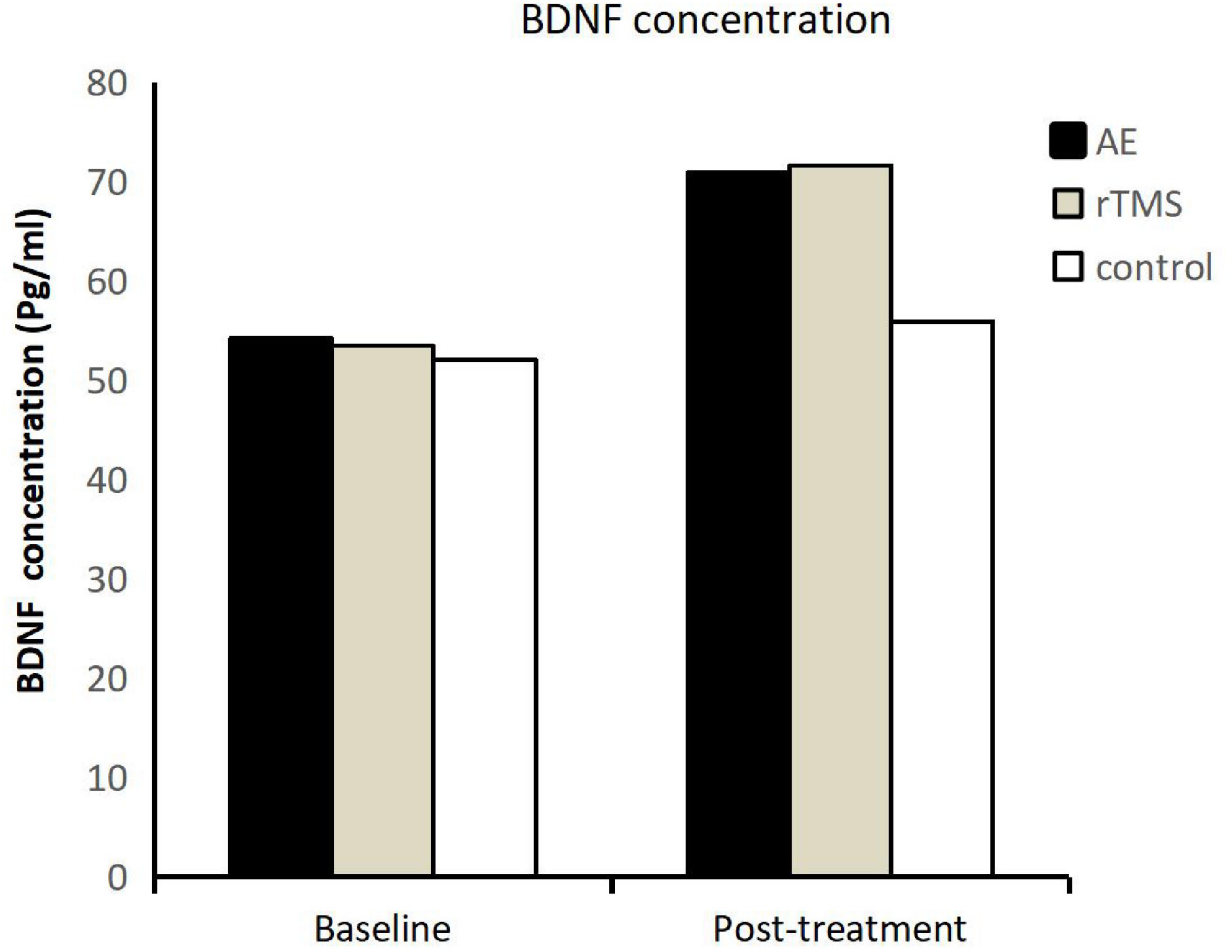
Brain-derived neurotrophic factor (BDNF) levels in three groups of adolescents with depression before and after intervention.

After exploring the impact of different intervention methods on serum serotonin (5-HT) levels, this study delved into the investigation of brain-derived neurotrophic factor (BDNF) levels in the serum of patients. The findings revealed a consistent upward trend in serum BDNF levels in adolescents with depression undergoing antidepressant treatment and interventions, mirroring the pattern observed for 5-HT (as illustrated in Figure 4).

Prior to intervention, there were no significant differences in serum BDNF levels among the groups (*F* = 0.010, *p* = 0.990). The pre-intervention BDNF concentrations were 320.84 ± 112.60 pg/ml for the exercise group, 321.99 ± 102.30 pg/ml for the rTMS group, and 316.26 ± 127.79 pg/ml for the control group.

Post-intervention, the BDNF concentrations increased to 446.51 ± 108.47 pg/ml for the exercise group, 449.11 ± 84.24 pg/ml for the rTMS group, and 341.73 ± 144.51 pg/ml for the control group. The time main effect for BDNF demonstrated a significant difference (*F* = 19.825, *p* < 0.001, η^2^ = 0.321), indicating that patients undergoing inpatient antidepressant treatment, regardless of the intervention type, experienced an elevation in serum BDNF levels. Furthermore, the interaction effect between time and group for BDNF was not significant, and the simple main effect on the group was also not significant (*F* = 1.860, *p* = 0.168, η^2^ = 0.081). Further comparisons among the groups revealed that although the exercise and rTMS groups showed a substantial increase in BDNF levels, the observed differences were not statistically significant (*p* > 0.05).

## 4 Discussion

### 4.1 Impact of aerobic exercise and rTMS on depression symptoms and cognitive abilities in adolescents with depression

This study discovered that supplementing a 4-weeks aerobic exercise intervention alongside pharmacological treatment improves the clinical effectiveness of antidepressant therapy. It markedly ameliorates depressive symptoms, reduces depression levels, and enhances cognitive functions in patients with depression. Concerning the enhancement in cognitive functions, the study unveiled that a 4-weeks aerobic exercise intervention significantly improves patients’ attention, attention shift, learning, working memory, and cognitive flexibility.

Previous research has also delved into the effects of aerobic exercise on alleviating depression and enhancing cognitive functions. Some studies have indicated that aerobic exercise can enhance visual learning and memory in individuals with depression, with the maximum benefits for executive function improvement observed after 6–8 weeks of aerobic exercise (Radovic et al., 2017, 2018; Bursnall, 2014; Wegner et al., 2020; Vancampfort et al., 2020; Schuch et al., 2016; Carter et al., 2016; Toups et al., 2017; Bailey et al., 2018).

In contrast to prior studies, the exercise intervention in this research involved supervised moderate-intensity pedaling exercise using a stationary bicycle, conducted four times a week for 30 min each session over a continuous 4-weeks period. Unlike previous research, where typically aerobic exercise interventions of 6 weeks or more were effective in improving depressive symptoms, this study revealed that a 4-weeks exercise intervention, combined with pharmacological treatment, significantly enhanced patients’ cognitive functions, including attention, attention shift, learning, working memory, and cognitive flexibility. The effective duration for aerobic exercise intervention, which was previously considered effective only after 6 weeks or more, was shortened to 4 weeks in this study. Apart from the influence of pharmacological intervention, the significant role of supervised exercise, possibly guided by a professional, could contribute to this notable effect.

### 4.2 Impact of aerobic exercise and rTMS on serum 5-HT and BDNF levels in adolescents with depression

In this study, we utilized the concentrations of serotonin (5-HT) and brain-derived neurotrophic factor (BDNF) as indicators to investigate the mechanisms of depression. The neurobiological mechanisms associated with depression primarily encompass endocrine mechanisms, neurotrophic factor mechanisms, anti-inflammatory mechanisms, HPA axis mechanisms, central nervous system structural morphology, genetic mechanisms, and gut microbiota. In this research, the concentration of 5-HT was chosen to reflect the endocrine mechanism of depression, while the concentration of BDNF was selected to represent the neurotrophic factor mechanism.

The results of our study reveal a significant increase in serum levels of both 5-HT and BDNF in adolescents with depression after aerobic exercise and rTMS interventions. This finding aligns with previous research, indicating that both aerobic exercise and rTMS can enhance the concentration of serotonin and neurotrophic factors in individuals with depression. However, there are distinct effects among the groups concerning the impact on 5-HT and BDNF. Specifically, the aerobic exercise and rTMS groups demonstrated a significant difference from the control group in elevating 5-HT concentration, while there was no significant difference in BDNF concentration between the aerobic exercise and rTMS groups compared to the control group.

The results regarding BDNF may appear contradictory to earlier findings (i.e., significant differences between aerobic exercise, rTMS, and the control group in influencing depression symptoms and cognitive function). However, this can be reasonably explained. Aerobic exercise, rTMS, and conventional inpatient treatment are all effective in improving depression symptoms and enhancing cognitive abilities. However, the extent of relief and improvement may vary, suggesting that these interventions operate through different mechanisms to ameliorate depression. BDNF reflects the neurotrophic factor mechanism. The conclusions drawn from this study suggest that aerobic exercise, repetitive transcranial magnetic stimulation, and conventional inpatient treatment (including a range of inpatient treatments, such as medication) may not differ significantly in their impact on the neurotrophic factor mechanism in adolescents with depression. However, concerning changes in 5-HT, aerobic exercise and rTMS exhibit significant differences compared to conventional inpatient treatment. This outcome implies that aerobic exercise and rTMS may be more effective than conventional inpatient treatment in improving the neuroendocrine mechanism of depression in adolescents. The improvements in neuroendocrine function induced by aerobic exercise and rTMS may surpass those achieved through conventional inpatient treatment.

The results of this study not only affirm the effectiveness of exercise and rTMS in improving depression levels and cognitive abilities in adolescents with depression but also reconfirm their significant impact on neurotrophic factor and endocrine mechanisms. However, there are subtle differences between the findings of this study and some previous research. In particular, the concentration range and post-intervention increase in serum 5-HT and BDNF levels in adolescents in our study may differ from those in other studies. For instance, at baseline, the concentrations of BDNF and 5-HT in the serum of patients in our study were relatively high compared to studies targeting elderly individuals with depression, postpartum depression patients, or studies without age controls but only controlling for the severity of depression. This divergence might be attributed to the unique characteristics of the adolescent population selected for this study.

Why do adolescents with depression exhibit higher baseline levels of 5-HT and BDNF compared to other age groups? This study posits several possible reasons. Firstly, previous research indicates that individuals with depression may experience multiple depressive episodes throughout their lives (Hammar et al., 2022). Depressive episodes induce changes in various depression-related mechanisms, such as disruptions in endocrine balance leading to decreased serotonin levels and alterations in neurotrophic factor levels, such as reduced BDNF concentrations in the serum. After treating depressive episodes, neurotransmitters, hormones, receptors, and other altered components do not return to baseline levels; instead, some damage persists. The neurotoxicity inherent in depression can stimulate recurrent depressive episodes, accumulating the effects of reductions in neurotransmitter and hormone levels. Individuals with depression who have experienced multiple recurrences may exhibit lower concentrations of BDNF and 5-HT in the serum compared to those with single or fewer recurrences. Additionally, since the onset of depression often occurs during adolescence (Ssegonja et al., 2019; Johnson and Taliaferro, 2011), most adolescents with depression may be experiencing depression for the first time or have fewer episodes of recurrence. They likely have fewer instances of depression than elderly or adult patients with depression, indicating that their decrease in BDNF and 5-HT levels would be less than in other age groups, resulting in higher baseline levels. In our study, the increase in BDNF concentration from baseline reached up to 40%, indicating a substantial increase. This may be because the participants in this study were adolescents with depression. The adolescent stage is unique; individuals at this stage are highly susceptible to external environmental changes (Shorey et al., 2022) and more sensitive in their responses to the external environment. Therefore, external stimuli may elicit greater responses in them compared to other age groups, explaining why, after exercise and rTMS interventions, adolescents with depression in our study showed a larger increase in serum concentrations of 5-HT and BDNF compared to other studies.

Moreover, different intervention methods and durations may have varying effects on depression and cognitive abilities. The exercise intervention and rTMS protocols chosen for this study, compared to those in other studies, might be considered preferable for adolescents with depression. These interventions demonstrated a more pronounced effect in alleviating depressive symptoms and improving cognitive abilities, as reflected in the substantial increase in 5-HT and BDNF concentrations. Although the concentrations of these two indicators were higher than those in patients with depression in other age groups and exhibited a significant increase after 4 weeks of intervention, they still fell considerably below the normal reference range for BDNF and 5-HT. There might be several reasons for this observation. The short duration of the 4-weeks intervention might have limited the manifestation of the effects in blood indicators, as these indicators represent the outcomes of various neuroendocrine systems working in the body, and the effects of interventions may gradually appear over time. Another possibility is that both exercise and rTMS cannot cure depression but only alleviate depressive symptoms to enable patients to maintain normal social activities. As a result, these blood indicators may not increase to normal levels. In the future, refining the exercise intervention methods and using 5-HT and BDNF levels as indicators may help identify the optimal exercise intervention protocol.

The study also found that the concentrations of brain-derived neurotrophic factor (BDNF) and serotonin (5-HT) in the serum were significantly higher in patients after exercise and rTMS interventions compared to the control group, with no significant difference between the two intervention groups. Through the application of aerobic exercise and repetitive transcranial magnetic stimulation (rTMS) interventions on adolescents with depression, combined with the analysis of physiological and biochemical indicators of depression, and considering the results of our previous analysis on the impact of aerobic exercise and rTMS on depressive symptoms and cognitive abilities in adolescents with depression, we can confidently conclude that aerobic exercise intervention can serve as an effective alternative to rTMS intervention. This consistent conclusion is supported by the observed improvements in both physiological and biochemical indicators, reinforcing the notion that aerobic exercise can produce comparable effects to rTMS in mitigating depression-related symptoms and enhancing cognitive abilities in adolescents with depression. This finding has significant implications for the clinical management of adolescent depression, providing a non-invasive and potentially more accessible intervention option in the form of aerobic exercise.

Exercise is considered a safe intervention method, as evidenced by the absence of any reported side effects. In addition to the observed increase in serum BDNF and 5-HT levels in adolescents after exercise, the mechanisms through which exercise improves depression are multifaceted. Existing research suggests that exercise can enhance the function of the hypothalamic-pituitary-adrenal (HPA) axis, contributing to the amelioration of depression (Rethorst et al., 2015). Moreover, exercise induces a rapid elevation in the muscle protein interleukin-6 (IL-6), leading to increased secretion of other anti-inflammatory factors. This, in turn, inhibits the production of pro-inflammatory cytokines such as TNF-α, IL-6, and IL-8, while elevating the baseline level of TNF-α. The downregulation of pro-inflammatory cytokine transcription, increased sensitivity of glucocorticoid receptors, and normalization of HPA feedback regulation are among the anti-inflammatory effects of exercise. Furthermore, exercise has been linked to improvements in glucose tolerance, thereby addressing sleep issues in individuals with depression (Rethorst et al., 2015). Poor glucose tolerance is associated with reduced sleep quality, and exercise can enhance glucose tolerance to ameliorate sleep problems in depression. The anti-inflammatory effect of exercise, in part, contributes to the improvement of sleep mechanisms. Aerobic capacity has been identified as an indicator of overall health, with exercise promoting aerobic capacity to enhance physical well-being. Increased physical fitness resulting from exercise leads to an augmentation of gray matter in the brain, contributing to the alleviation of depression.

Additionally, exercise has been shown to counteract the reduction in hippocampal volume associated with depression. Depression is linked to a decrease in hippocampal volume, and exercise can lower the apoptosis rate of hippocampal cells, thereby increasing hippocampal volume and alleviating depression. Growth factors in the hippocampus, such as vascular endothelial growth factor (VEGF) and insulin-like growth factor-1 (IGF-1), play crucial roles in cognition and growth regulation. Exercise modulates the levels of these factors in the central nervous system, promoting neurogenesis, survival, and differentiation of hippocampal neurons, ultimately increasing hippocampal volume. This mechanism contributes to the enhancement of neural plasticity and the improvement of depression (Peng et al., 2023).

Exercise-induced elevation of BDNF levels has been associated with improved memory function in depression. The increase in BDNF levels can enhance memory, contributing to the improvement of hippocampal function in individuals with depression. Studies have indicated that 12 months of moderate-intensity aerobic exercise can increase hippocampal volume by approximately 2%, demonstrating the effectiveness of aerobic exercise in enhancing hippocampal structure and function. Depression is also correlated with the gray matter volume of the prefrontal cortex (PFC), with a reduction in gray matter volume being a significant physiological marker in individuals with depression. Severe depression is characterized by a decrease in gray matter volume, affecting the volumes of the prefrontal cortex, cingulate gyrus, and temporal lobe (particularly a significant decrease in gray matter volume in the frontal and marginal brain regions). Exercise interventions of 6 months with moderate intensity have been shown to significantly increase gray matter volume in the prefrontal cortex, including the frontal cortex, frontal pole, paracentral lobule, precentral gyrus, and posterior cingulate gyrus. The volume of the superior frontal cortex remained almost unchanged. By enhancing physical fitness, exercise increases prefrontal cortex gray matter volume, mitigating depression. Exercise effectively regulates concentrations of neurotrophic factors, hormone levels, specific morphological structures in the central nervous system, and the release of pro-inflammatory cytokines. It induces effective stimulation of hippocampal neurogenesis within the central nervous system. Therefore, exercise is a highly recommended and accessible intervention for alleviating depression in adolescents, offering a range of physiological and psychological benefits.

In the context of repetitive transcranial magnetic stimulation (rTMS) intervention, despite abundant research demonstrating its relative safety and benefits for children and adolescents with major depressive disorder (MDD) (Qiu et al., 2023; Hett et al., 2021; Bejenaru and Malhi, 2022), as well as its capacity to alleviate symptoms of adolescent depression and being a promising treatment method for psychiatric disorders in this demographic, concerns persist among depressed adolescents and their parents regarding the safety of transcranial magnetic stimulation. There is a lack of understanding about the mechanisms and impacts of transcranial magnetic stimulation on the human body, and there is reluctance to permit depressed adolescents to undergo transcranial magnetic stimulation therapy due to fears of potential brain damage. Beyond safety concerns, the use of rTMS may also impose additional economic burdens. Nevertheless, rTMS treatment is deemed essential, especially as an effective intervention for treatment-resistant severe depression (Corlier et al., 2020; Oberman et al., 2021) and a hopeful alternative for patients who do not respond to traditional medication. Consequently, the aerobic exercise intervention discovered in this study can serve as a viable alternative to rTMS in treating adolescent depression, thereby alleviating many of the associated concerns. This finding provides valuable insights for formulating clinical combined intervention prescriptions, highlighting aerobic exercise intervention as an effective alternative to rTMS in the treatment of depression among adolescents.

### 4.3 Limitations and strengths

This study has several limitations that should be considered. First, the specific types and dosages of SSRIs were not controlled, which may introduce confounding. Second, the lack of a sham rTMS control group makes it difficult to fully isolate the specific neurophysiological effects of rTMS from placebo effects. Third, the 4-weeks intervention period may be too short to observe long-term or maximal neurotrophic changes. Finally, the sample consisted exclusively of female inpatients, which may limit the generalizability of the findings. Despite these limitations, this study also has notable strengths, including a randomized controlled design, the use of both clinical and neurobiological outcome measures, and a direct comparison between two promising non-pharmacological interventions in a well-defined adolescent clinical population.

## 5 Conclusion

For adolescents with depression, incorporating a 4-weeks intervention comprising aerobic exercise and rTMS in conjunction with inpatient treatment produces superior clinical outcomes compared to pharmacotherapy alone. Both adjunctive interventions of aerobic exercise and rTMS significantly alleviate clinical symptoms, diminish depression levels, and enhance cognitive functions such as learning, working memory, and cognitive flexibility in adolescents with depression. Furthermore, these interventions elevate serum concentrations of serotonin (5-HT) and brain-derived neurotrophic factor (BDNF) with comparable efficacy between the two approaches.

## Data Availability

All relevant data supporting the conclusions of this study have been fully included in the main text of the manuscript and the supplementary materials. For additional raw data of this study or detailed explanatory materials on relevant experimental methods, please contact the first author, YT (Email: 840835737@qq.com), via a reasonable academic request.
